# BAFF blockade in experimental autoimmune encephalomyelitis reduces inflammation in the meninges and synaptic and neuronal loss in adjacent brain regions

**DOI:** 10.1186/s12974-023-02922-7

**Published:** 2023-10-07

**Authors:** Kanak Gupta, Ajay Kesharwani, Steven Rua, Saumitra Sen Singh, Catherine Siu, Larissa Jank, Matthew D. Smith, Peter A. Calabresi, Pavan Bhargava

**Affiliations:** Division of Neuroimmunology and Neurological Infections, Department of Neurology, Johns Hopkins Hospital, Johns Hopkins University School of Medicine, Pathology Building, 600 N. Wolfe St., Pathology 627, Baltimore, MD 21287 USA

**Keywords:** BAFF, BAFF antagonist, Anti-BAFF antibody, Meningeal inflammation, Multiple sclerosis, EAE

## Abstract

Multiple sclerosis (MS) has traditionally been viewed as a chronic inflammatory disease affecting the white matter of the central nervous system. However, over the past two decades, increasing evidence has highlighted the role of gray matter pathology in MS-related disability. Numerous studies have linked the presence of leptomeningeal inflammation to a more severe disease course, underscoring its potential importance as a driver of gray matter pathology in MS. The major components of leptomeningeal inflammation include T cells, B cells, macrophages, follicular dendritic cells, and plasma cells. Since BAFF [B cell-activating factor of the tumor necrosis factor (TNF) family] promotes B cell survival and maturation and is a co-stimulator of T cells, we used anti-BAFF antibody 10F4 as a BAFF antagonist to study its effect on meningeal inflammation and adjacent brain regions in a relapsing–remitting PLP-EAE (rr-EAE) model of multiple sclerosis in SJL/J mice. rr-EAE mice were treated either with anti-BAFF antibody 10F4 or with IgG control antibody. We performed ultra-high field (11.7 T) MRI to identify areas of meningeal inflammation and track them over time in both treatment groups. We also performed histopathological analysis in brain sections of these mice to study the effects of the BAFF antagonist on leptomeningeal inflammation, and hippocampal and cortical neurons and synapses. We observed that BAFF antagonist treatment reduced B cells, T cells, and myeloid cells in regions of meningeal inflammation. Additionally, we noted that BAFF treatment protected against EAE-induced synaptic and neuronal loss in the adjacent cortex and in the CA1, CA3, and dentate gyrus regions of the hippocampus likely due to its effects on meningeal inflammation.

**Supplementary Information:**

The online version contains supplementary material available at 10.1186/s12974-023-02922-7.

## Introduction

Multiple Sclerosis is a chronic autoimmune disease characterized by episodes of neurological dysfunction such as loss of vision, ataxia, sensory loss, and limb weakness. Pathologically, MS is identified by plaque-like brain lesions caused by demyelination and inflammation [1]. Relatively recent studies have also identified the presence of leptomeningeal inflammation in all forms of MS with a greater proportion affected in progressive disease [2–5]. These regions of inflammation were found to consist of aggregates of inflammatory cells—B cells, T cells, plasma cells, and dendritic cells [3]—and have been linked to earlier onset and greater severity of disease [6]. Particularly in the case of SPMS, B cell follicle-like structures were found adjacent to subpial cortical lesions and correlated with greater cortical demyelination and microglial activation [6, 7]. Reduction of B cell infiltration into the meninges, therefore, may lead to amelioration of MS severity.

BAFF (B cell-activating factor of the TNF family) presents itself as a potential therapeutic target in achieving this goal. BAFF and its structural homologue APRIL (A-proliferation-inducing ligand) are inflammatory cytokines involved in the survival, maturation, and proliferation of B cells, and the stimulation of T cells [8]. Dysregulation of BAFF and APRIL has been implicated in the pathogenesis of many immune disorders, with a recent study showing elevated levels of both in the cerebrospinal fluid (CSF) of patients with untreated MS [8, 9]. An increase in BAFF expression, along with that of CXCL13 (a cytokine involved in B cell recruitment), was also noted in the CNS of mice with relapsing–remitting and chronic-relapsing EAE (experimental autoimmune encephalomyelitis) in the presence of leptomeningeal inflammation [10]. In the CNS, BAFF can be endogenously produced by astrocytes and has been found to be upregulated close to BAFF-R and TACI expressing immune cells in MS lesions [11]. Moreover, recent studies have shown that BAFF stimulates Nogo receptors, NgR1 and NgR3, localized on B cells found in leptomeningeal infiltrates [12, 13]. Given this understanding of the role of BAFF in neuroinflammation and degeneration, BAFF blockade appears to be a promising therapeutic strategy for MS.

Interestingly belimumab, an FDA-approved monoclonal antibody medication, is utilized in the management of specific autoimmune disorders, notably systemic lupus erythematosus (SLE) and lupus nephritis and operates by binding to and neutralizing BAFF [14, 15]. This has not been tested in MS or other neuroimmunological diseases.

In this study, we examined the effects of the anti-BAFF antibody 10F4 (GlaxoSmithKline, Middlesex, UK), the murine analog of belimumab, on the composition and size of regions of leptomeningeal inflammation (LMI), and on the glial cell and neuronal populations in the hippocampus and cortex adjacent to LMI, in a relapsing–remitting EAE mouse model. The BAFF antagonist antibody utilized in this study binds with BAFF to prevent interaction with its receptors [16]. In the current study, we studied the effects of BAFF antagonism on leptomeningeal inflammation by means of immunohistopathology and MRI imaging. Fluorescent staining of post-mortem mouse brains with intact meninges was used to characterize the cellular composition of the leptomeningeal lymphoid aggregates and changes in adjacent grey matter. Based on previous studies, we also found in vivo contrast-enhanced MRI imaging to be a reliable marker of leptomeningeal inflammation—with contrast enhancements in the leptomeninges corresponding with regions of lymphoid aggregations [17–21]. Therefore, we used MRI data collected at multiple time points to calculate the volume of regions of leptomeningeal enhancement (LME) in SJL/J mice with rr-EAE and analyzed longitudinal changes in response to the treatment.

Here, we demonstrate that BAFF blockade led to reduced infiltration of B cells, T cells, and myeloid cells into the leptomeninges. We also observed protection of neuronal and synaptic density in the adjacent cortex and the hippocampus in anti-BAFF treated EAE mice.

## Methods

### Mice

Female SJL/J mice were purchased from Jackson Laboratories. All mice were kept in barrier rooms under 12-h light/dark cycle, fed ad libitum, at the federally approved animal care facility at Johns Hopkins University in accordance with the Institutional Animal Care and Use Committee. 9- to 12-week-aged female mice were used in all the experiments and were housed in the animal facility at least one week before the start of experiments.

### Induction of relapsing remitting EAE and scoring in SJL/J mice

Female SJL/J mice were immunized as described previously [17]. Briefly, mice were immunized subcutaneously on day 0 in the left and right flank with 100 µg of PLP_139-151_ peptide with complete Freund’s adjuvant containing 4 µg/ml *Mycobacterium tuberculosis* H37RA (Difco Laboratories). Mice were weighed, and clinical scores were recorded in a blinded manner. Clinical scoring was performed using the following scale in increments of 0.5: 0, normal; 1, limp tail; 2, hind limb weakness; 3, hind limb paralysis; 4, hind and forelimb weakness; and 5, death.

### Imaging of meningeal disease

MRIs were conducted on all immunized mice at the end of week 6 post-immunization. Mice without visible leptomeningeal enhancement (LME) on MRIs were excluded from the experiment, while LME-positive mice were scanned again at week 8 and week 10 post-immunization to monitor disease progression in treatment and control groups. While some of the excluded mice may have developed LME later in the course of their disease, since MRI LME changes over the course of the study were a major readout, we excluded mice without visible LME at baseline since they would not have a datapoint to track over the following scans and may not have subsequently developed LME. MRI scans were conducted following a previously defined protocol [19] using a horizontal 11.7 T scanner (Bruker BioSpin) with a triple-axis gradient system (maximum gradient strength = 740 mT/m), 72 mm volume transmit coil. A 4-channel receive-only phased array coil was used to image the mouse brain. 15 min before imaging, 0.1 ml diluted Magnevist^®^ (gadopentetate dimeglumine, Bayer HealthCare LLC, 1:8 with PBS) was injected intraperitoneally. Mice were anaesthetized with isoflurane together with mixed air and oxygen (2:1 ratio) during the imaging and respiration was monitored via a pressure sensor and maintained at 20–60 breaths/min. T2-weighted image sequences, with 30 axial slices of 0.5 mm thickness to cover the whole brain, were acquired using a rapid acquisition with refocused echoes (RARE) sequence with the parameters: echo time/repetition time = 30/5000 ms; echo train length = 8; field of view = 15 mm × 15 mm; matrix size = 192 × 192, and a signal average of 4. 15 T1-weighted axial slices to cover the forebrain were acquired using a spin echo sequence with the same parameters as the T2-weighted images except echo time/repetition time = 9/300 ms, matrix size = 128 × 128. FLAIR images were then acquired. The RARE sequence was used with an inversion pulse to obtain 15 axial slices of 0.5 mm thickness with the same matrix size and imaging geometry as the T1-weighted images, with parameters echo time/repetition time = 20/3000 ms, steady-state inversion time = 1000 ms after a sech adiabatic inversion pulse.

**Fig. 1:**
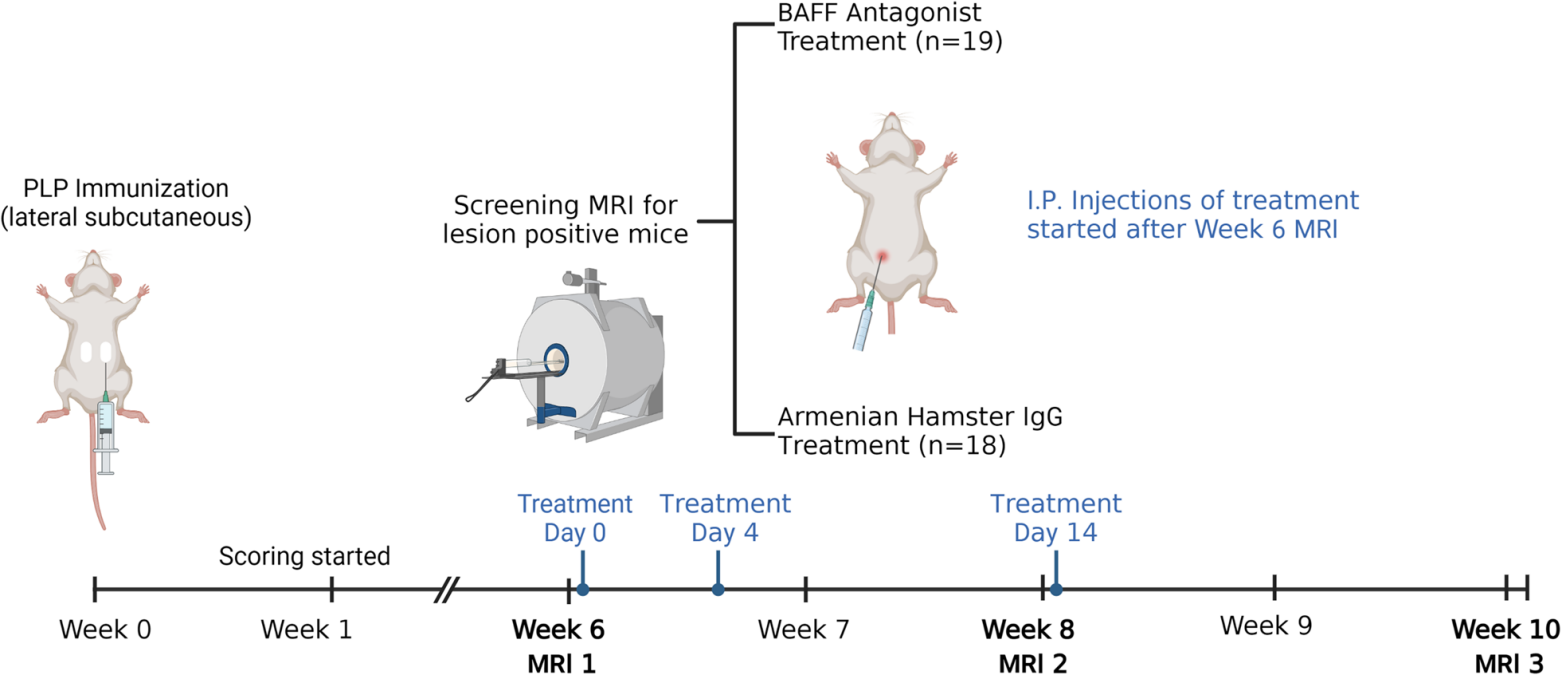
**Experimental design.** 9- to 12-week-old female SJL/J mice were immunized subcutaneously with PLP_139-151_ with complete Freund’s adjuvant to induce rr-EAE. Clinical scoring was conducted starting one-week post-immunization. MRIs were conducted on all mice at the end of Week 6 post-immunization to screen for leptomeningeal enhancement (LME). Mice found positive for leptomeningeal enhancement (LME) were randomized to either BAFF antagonist (10F4) or IgG control treatment and injected at three time points—Day 0, Day 4, and Day 14 after Week 6 MRI. Daily clinical scoring and subsequent MRIs were conducted at the end of Week 8 and Week 10 post-immunization to monitor disease progression over time. Mice were killed the day after the Week 10 MRIs. Skulls were collected and decalcified using 14% EDTA for 2 weeks and used for histopathological analysis (created with BioRender.com).

Scans were analyzed to identify areas of meningeal contrast enhancement in the hippocampal fissure and cortical surface. Areas of meningeal contrast enhancement were manually outlined and measured (in pixels) on each of the 15 slices of the FLAIR axial MRI images, using image analysis software Fiji. Contrast enhancements in separate regions of the brain were treated as separate LME regions (Additional file 1: Fig. S2). The volume of each LME region was inferred by calculating the volume of the frustum (irregular cylinder) between aligning areas of high-contrast on consecutive slices $\left[V= \frac{h}{3 }\left({A}_{1}+{A}_{2}+ \sqrt{{A}_{1}{A}_{2}}\right)\right]$, where A_1_ = area of high-contrast LME in slice *n*, A_2_ = area of high-contrast LME in slice *n* + *1*, and *h* = slice thickness = 1.89 pixels]. The sum of all frustums of contrast-enhanced areas was used to calculate the Total LME Volume (in voxels) for each MRI. All quantifications were performed by at least two independent examiners (KG, AK, SR, PB) and their volume quantifications were then averaged (Fig. 1).

### BAFF antagonist treatment

Mice with visible contrast enhancement in Week 6 MRIs were randomized into two treatment conditions: anti-BAFF antibody (10F4; GlaxoSmithKline, Middlesex, UK) or with IgG control (Armenian Hamster Immunoglobulin G Isotope Control; Leinco Technologies, Inc.). Mice were matched by similar severity of illness as indicated by their clinical EAE scores. The mice were I.P. injected 0.1 ml of their respective treatments at concentrations of 1 mg/ml in PBS (i.e., 5 mg/kg mouse weight) [22, 23] on Days 0, 4, and 14 post-Week 6 MRI. Effects of the treatment were observed by analyzing MRIs conducted at following timepoints (Week 8 and Week 10) and post-mortem histopathology.

### Immunohistochemistry to characterize anti-BAFF effect on meningeal inflammation

Immunohistochemical analyses were performed as described in [19]. Briefly, mice were deeply anesthetized with isoflurane. After anesthesia, mice underwent intracardiac perfusion with 4% paraformaldehyde. The skull was separated, and the soft tissues were removed from it. The skull was then transferred for decalcification in 14% EDTA at pH 7.6. Skulls were weighed daily for approximately 2 weeks to determine the maximum decalcification. After decalcification, the skulls were transferred to 30% sucrose for 48 h. The skulls were embedded in O.C.T. before snap-freezing. 10-µm-thick cryo-sections were made onto glass slides (SuperFrost PlusTM, Fisherfrom) from brain tissue for immunohistochemical study.

For immunolabelling, the brain tissue sections on the glass slides were permeabilized with 0.4% Triton™ X-100 in PBS. The optional antigen retrieval was performed with heated citrate buffer (pH 6) for 8–10 min before blocking. After antigen retrieval, the sections were blocked with 5% normal goat serum in 0.4% Triton™ X-100 in PBS for 1 h at room temperature. Following the blocking, the sections were incubated with primary antibody in appropriate dilution (Table 1) in blocking buffer containing 5% NGS, 0.1% Triton™ X-100 overnight at 4 °C. Next day, the sections were washed and incubated with appropriate secondary antibodies conjugated to Alexa™ fluorophores (1:1000, Invitrogen), in 5% NGS and 0.1% Triton™ X-100 for 1 h at room temperature followed by nuclei staining with Hoechst. A coverslip was mounted onto the sections using aqua poly/mount reagent (Polysciences, Warrington, PA, USA). The following antibodies were tested, B220, CD3, GFAP, Iba-1 and Mac2 (see list of antibodies in Table 1).

**Table 1:** Antibodies used for immunohistochemistry

Antibody	Source	Clone	Host	Dilution	Antigen retrieval
B220	Thermo Fisher Scientific	Mono; RA3-6B2	Rat	1:200	Yes
CD3	Agilent Dako	Poly	Rabbit	1:200	Yes
Iba-1	Wako	Poly	Rabbit	1:500	No
GFAP	Cell signaling	Mono	Mouse	1:500	No
Mac2	BioLegend	Mono; M3/38	Rat	1:200	Yes
NeuN	Millipore ABN91	Poly	Chicken	1:500	Yes
PSD95	Abcam 18,258	Poly	Rabbit	1:500	Yes

Images were captured using a Zeiss Axio Observer Z1 epifluorescence microscope and Axiovision software with the appropriate excitation and emission filters. Each slide was imaged with three sections from each group (anti-BAFF Vs vehicle-treated) and each panel was processed with ZEN lite (ZEISS) and National Institutes of Health ImageJ software (NIH, https://imagej.nih.gov/ij/). The quantification of B220, CD3, and Mac2 immunosignals within the meningeal inflammatory infiltrates and Iba-1 and GFAP immunosignals in the cortex region adjacent to leptomeningeal inflammation was performed using ImageJ. Raw-integrated density of B220, CD3, and Mac2, was normalized to the Raw-integrated density of DAPI to calculate the % B220, CD3, and Mac2 of DAPI in the meninges. To quantify GFAP, and Iba-1 in the cortex adjacent to or surrounding areas of meningeal inflammatory infiltrates, ROIs were made in the cortex region and raw integrated density was measured using ImageJ. Raw-integrated density was normalized to the area of the ROI to calculate Mean Integrated Density. Based on the normality of the data, a Welch’s t-test or a non-parametric Mann–Whitney *U*-test was used to calculate the statistical significance between anti-BAFF versus vehicle-treated group for all the antibodies.

To assess synaptic and neuronal changes in the CA1, CA3, and DG region of hippocampus, PSD95 and NeuN antibodies were used. The immunosignal of PSD95 and NeuN in the CA1, CA3, DG, and in the brain parenchyma adjacent to the leptomeningeal infiltrates was quantified by making separate ROIs. Identical ROIs were used across all brain sections for each region of the hippocampus and the Raw Integrated Density of NeuN and PSD95 were calculated using ImageJ. in the brain parenchyma adjacent to the leptomeningeal infiltrates, the number of NeuN-positive nuclei per µm^2^ was also manually counted using Zen 3.4 (blue edition, ZEISS). The collected data were tested for normality distribution, and based on the results of this analysis, a non-parametric Mann–Whitney *U*-test was applied to determine statistical significance. Overall, this analysis allowed us to gain a better understanding of the changes that occur in the hippocampus in response to the meningeal infiltrate.

### Statistical analysis

Comparisons for Total LME Volumes and clinical scores over time were conducted using a mixed-effects analysis. Histopathological data were compared using Welch’s t-test when data were normally distributed and Mann–Whitney *U*-test when they were not. The threshold for significance was *P < 0.05. All analyses were conducted using GraphPad Prism 9.4.1.

## Results

### BAFF inhibition alters meningeal infiltrate in rr-EAE mice

**Fig. 2:**
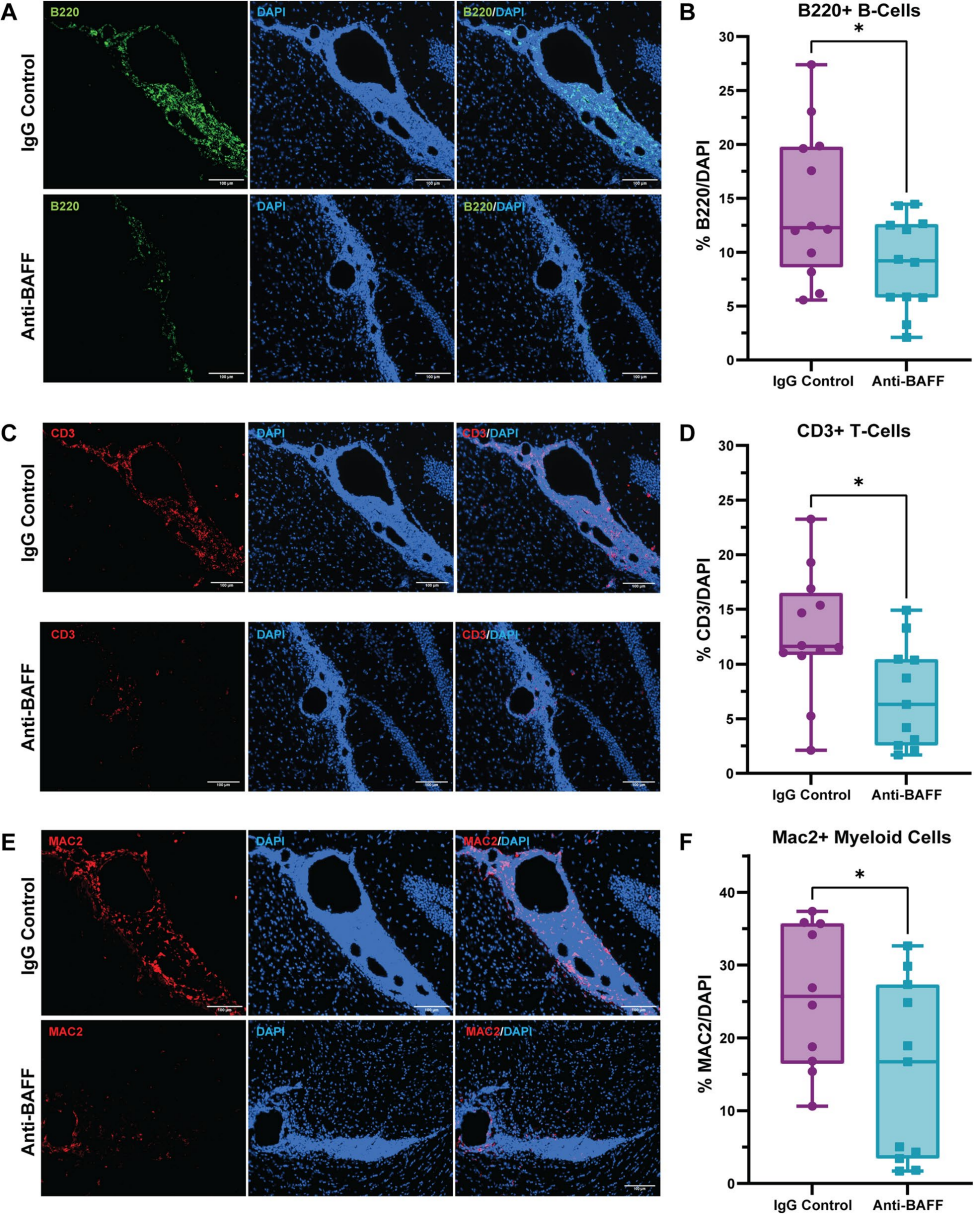
**Anti-BAFF antibody 10F4 reduced leptomeningeal inflammation detected through histopathology. A**, **B** On pathological evaluation at Week 10, we observed a decrease in the proportion of B cells within the meningeal infiltrate in the anti-BAFF antibody 10F4 group compared to the IgG control group. **C**, **D** Additionally, the treatment with the anti-BAFF antibody 10F4 led to a significant reduction in CD3 + T cells compared to control IgG-treated EAE mice. E, F We also found a significant difference in the proportion of myeloid cells (Mac2 +) between the anti-BAFF antibody 10F4 and IgG control group, with Mac2 + cells being less in the anti-BAFF treatment group. The number of EAE mice for the anti-BAFF antibody 10F4 group was 12 and for the IgG control group was 12. For B, D, F, data are shown as box plots, the center line indicates the median, the box indicates the 25th and 75th percentiles, the whiskers range, and the dots indicate all data points. The statistical analysis was conducted using two sample Welch’s t-test as data were normally distributed. *P < 0.05. Scale bars = 100 um

We utilized immunohistochemical staining to assess the effect of BAFF inhibition on the composition of meningeal infiltrates in mice with EAE. We noted a significant decrease in the proportion of B cells (B220+) within areas of meningeal inflammation in EAE mice treated with the anti-BAFF antibody in comparison to EAE mice treated with IgG control (Fig. 2A, B). We also observed a significant reduction in the proportion of CD3 + T-cells with anti-BAFF treatment (Fig. 2C, D). Additionally, together with B cells and T cells, the infiltration of Mac2 + myeloid cells in the meninges was also significantly reduced in EAE mice treated with anti-BAFF (Fig. 2E, F).

**Fig. 3:**
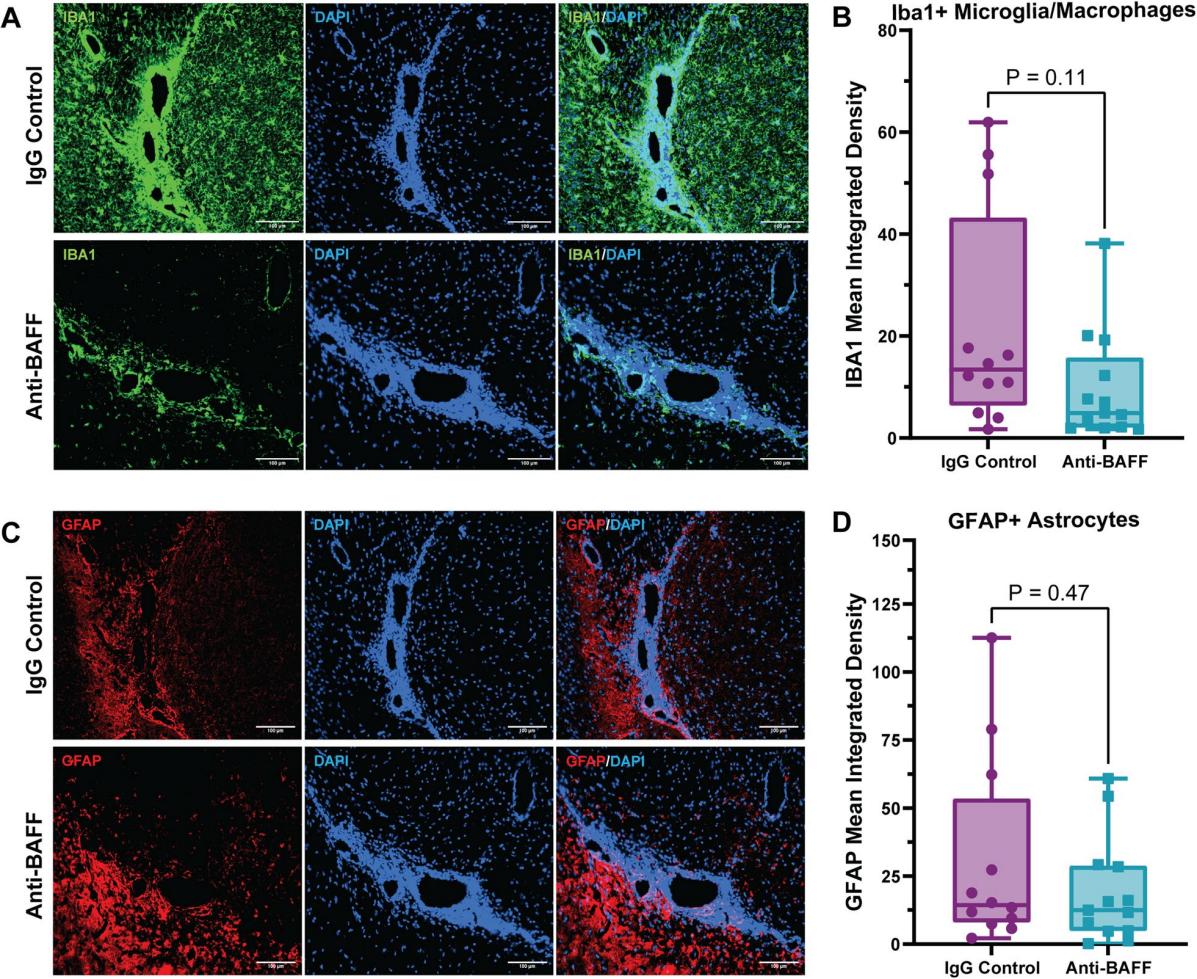
**Effects of anti-BAFF antibody 10F4 on glia in adjacent brain tissue.** **A**, **B** Histopathological immunofluorescence evaluation of IBA1 immunoreactivity revealed no significant change in the immunoreactivity of IBA1 in the cortex adjacent to the areas of meningeal inflammation in the anti-BAFF treatment group compared to the IgG control group. **C**, **D** No significant changes were observed in astrocytosis detected by GFAP immunoreactivity in the cortex surrounding the regions of meningeal inflammation. **B**, **D** Data were presented as box plots with the center line indicating the median, the box indicating the 25th and 75th percentiles, the whiskers indicating range, (min–max) and the dots representing all data points. The statistical analysis was conducted using Mann–Whitney U-tests, and *P*-values were derived from it. The significance level was set to *P < 0.05. The scale bars = 100 um

We observed a trend towards a decrease in the immunoreactivity of Iba-1 + microglia/macrophages in the cortex adjacent to the regions of meningeal inflammation, which may imply that the reduction in B cell and T cell infiltration in the meninges impacts microglia/macrophages in the adjacent cortex (Fig. 3A, B). However, we did not observe a change in astrocytosis, detected by GFAP immunoreactivity, in the cortex surrounding the regions of meningeal inflammation (Fig. 3C, D).

We also observed that BAFF inhibition with anti-BAFF antibody 10F4 did not produce a significant effect on rr-EAE clinical behavioral scores when compared with the IgG control-treated mice (Additional file 1: Fig. S1).

These results indicate that parenteral administration of anti-BAFF antibody impacted meningeal inflammation by modifying the composition of immune cells within the meningeal infiltrates.

### Effects of BAFF blockade on leptomeningeal contrast enhancement

**Fig. 4:**
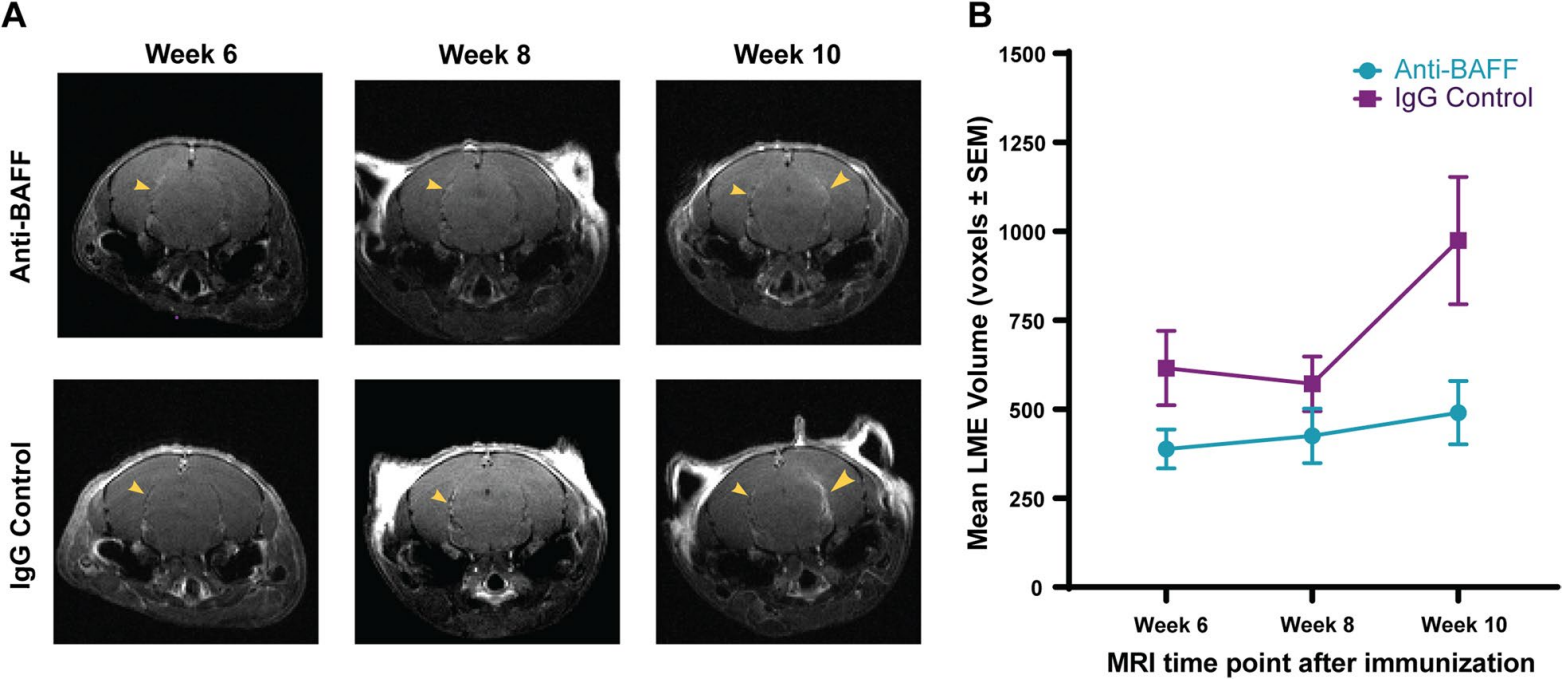
**Leptomeningeal inflammation quantification using contrast enhancement in ultra-high field MRI.** **A** Comparison of MRI scans across three time points (6, 8, and 10 weeks after immunization) in anti-BAFF treatment and IgG control groups. The yellow arrows identify areas of leptomeningeal contrast enhancement. **B** The total volume of leptomeningeal enhancement (LME) was averaged for each treatment group at each MRI time point. A mixed-effects analysis of the anti-BAFF treatment group (*n* = 19) and IgG control group (*n* = 18) did not show an interaction effect between time and treatment on mean volume of leptomeningeal contrast enhancements (*P* = 0.12)

In vivo MRI imaging was conducted to track changes in meningeal inflammation in mice over the course of 10 weeks, after immunization. We used contrast enhancement visible in the MRI images as a measure of meningeal inflammation. Contrast enhancement in the leptomeninges was clearly visible in FLAIR image sequences obtained in the MRIs and representative examples of the changes in contrast enhancement over the three MRI time points can be seen in Fig. 4A. Of the 60 immunized SJL/J mice, which were screened for meningeal contrast enhancement at the end of Week 6, 37 were positive for LME on the cortical surface or in the hippocampal fissure. Subsequent MRIs tracked changes in the volume of these LME or the appearance of new LME over the next 4 weeks. Week 8 scans did not show a significant increase in LME volumes. Week 10 scans show a steeper increase in the IgG control group as compared with the anti-BAFF treatment group. However, a mixed-effects analysis comparing the two groups showed that though time and treatment individually had significant effects (*P* = 0.019 and *P* = 0.013, respectively), there was no interaction effect between the two (*P* = 0.12) suggesting that anti-BAFF treatment did not significantly reduce leptomeningeal contrast enhancement compared to vehicle (Fig. 4B). However, the apparent trend towards increased LME volume in the IgG control group compared to the anti-BAFF treatment group indicates that perhaps a longer experiment duration with additional MRI time points may provide a clearer picture of whether this difference would be significant over a longer time period or with a larger sample size.

### BAFF blockade ameliorates synaptic and neuronal loss in hippocampus and cortex adjacent to meningeal inflammation in rr-EAE mice

Recent studies have shown that EAE leads to hippocampal neurodegeneration, which is characterized by decreased volume in the CA1 region and loss of GABAergic interneurons [24]. This neurodegeneration is also linked to dysfunction in learning and memory during spatial tasks that depend on the hippocampus. Additionally, EAE has been found to cause increased microglial activation in the hippocampus. The disease is also associated with increased apoptosis of neurons, interneurons, and astrocytes, and a significant decrease in synaptic puncta.

**Fig. 5:**
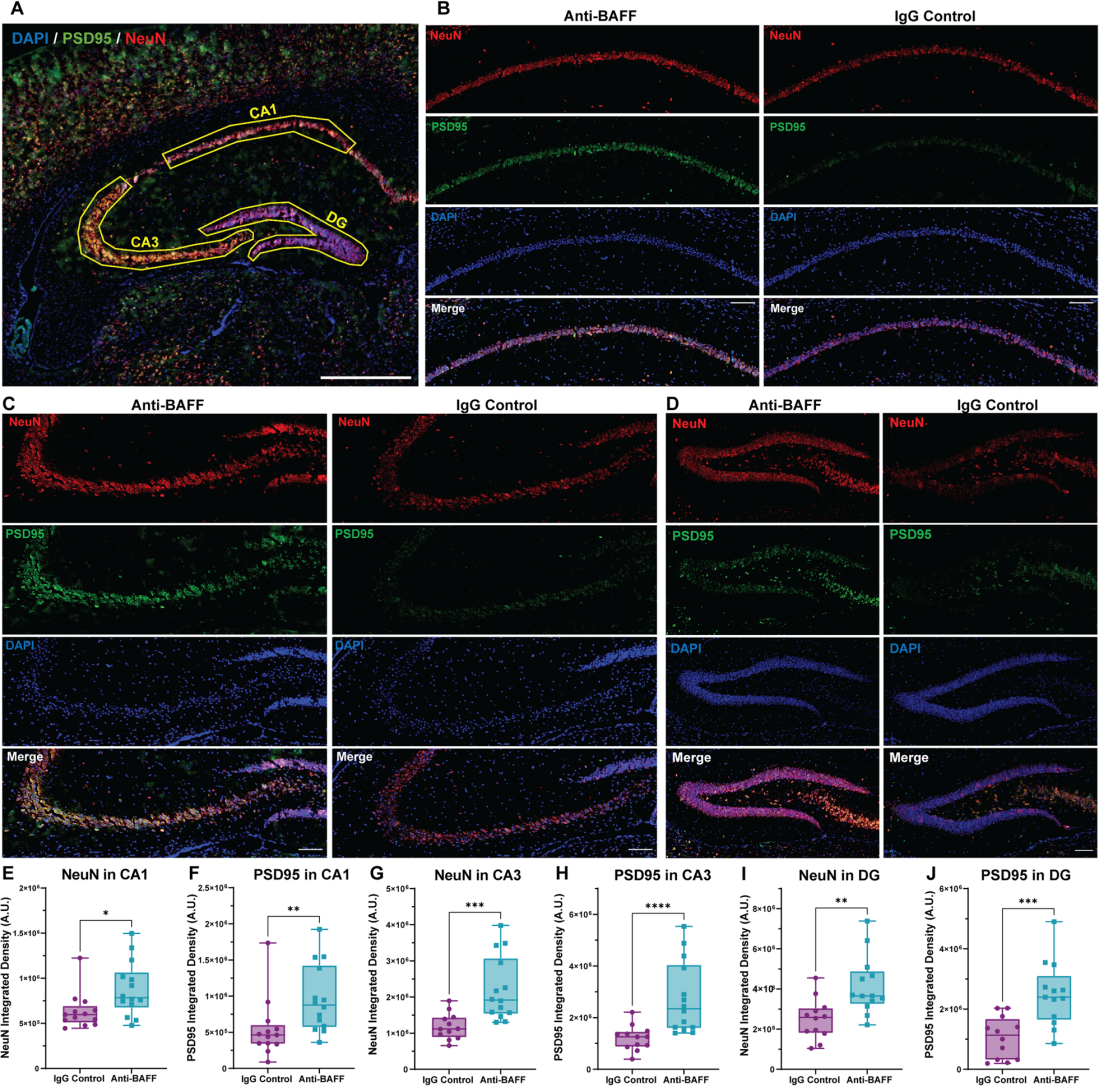
**BAFF blockade with anti-BAFF antibody 10F4 protects hippocampal synapses and neurons in rr-EAE.** **A** Overview of PSD95 (green), NeuN (red), and DAPI (blue) stained coronal section of brain from EAE mice showing the ROI of CA1, CA3, and dentate gyrus (DG) region used for quantification of PSD95 and NeuN integrated density to compare between the anti-BAFF antibody and control IgG-treated EAE mice. **B–D** Representative images of the CA1, CA3, and DG hippocampal region stained with PSD95 and NeuN from anti-BAFF treated, and IgG control-treated EAE mice brain coronal section. **E–J** Quantitative analysis shows significantly higher integrated density of PSD95 and NeuN in the CA1 region (**E**, **F**), CA3 region (**G**, **H**), and in the DG region (**I**, **J**) in anti-BAFF antibody-treated mice in comparison to the IgG control-treated mice. **E**–**J** Data were presented as box plots with the center line indicating the median, the box indicating the 25th and 75th percentiles, the whiskers indicating range, (min–max) and the dots representing all data points. The statistical analysis was conducted using Mann–Whitney *U*-tests. The significance level was set to *P < 0.05, **P < 0.01, ***P < 0.001. The scale bars for the images shown were 100 um. The number of EAE mice for the anti-BAFF antibody 10F4 group was 14 and for the IgG control group was 13

To investigate the potential involvement of BAFF in EAE-related synaptopathy, we performed immunolabelling to measure the expression of synaptic proteins in different hippocampus regions in brain sections from anti-BAFF antibody and IgG control-treated EAE mice (Fig. 5A). We used PSD95 and NeuN and measured the integrated density of these proteins in the CA1, CA3, and DG regions of the hippocampus and in the cortex adjacent to meningeal infiltrates. We also counted the NeuN + DAPI + puncta in the cortex adjacent to meningeal infiltrates.

**Fig. 6:**
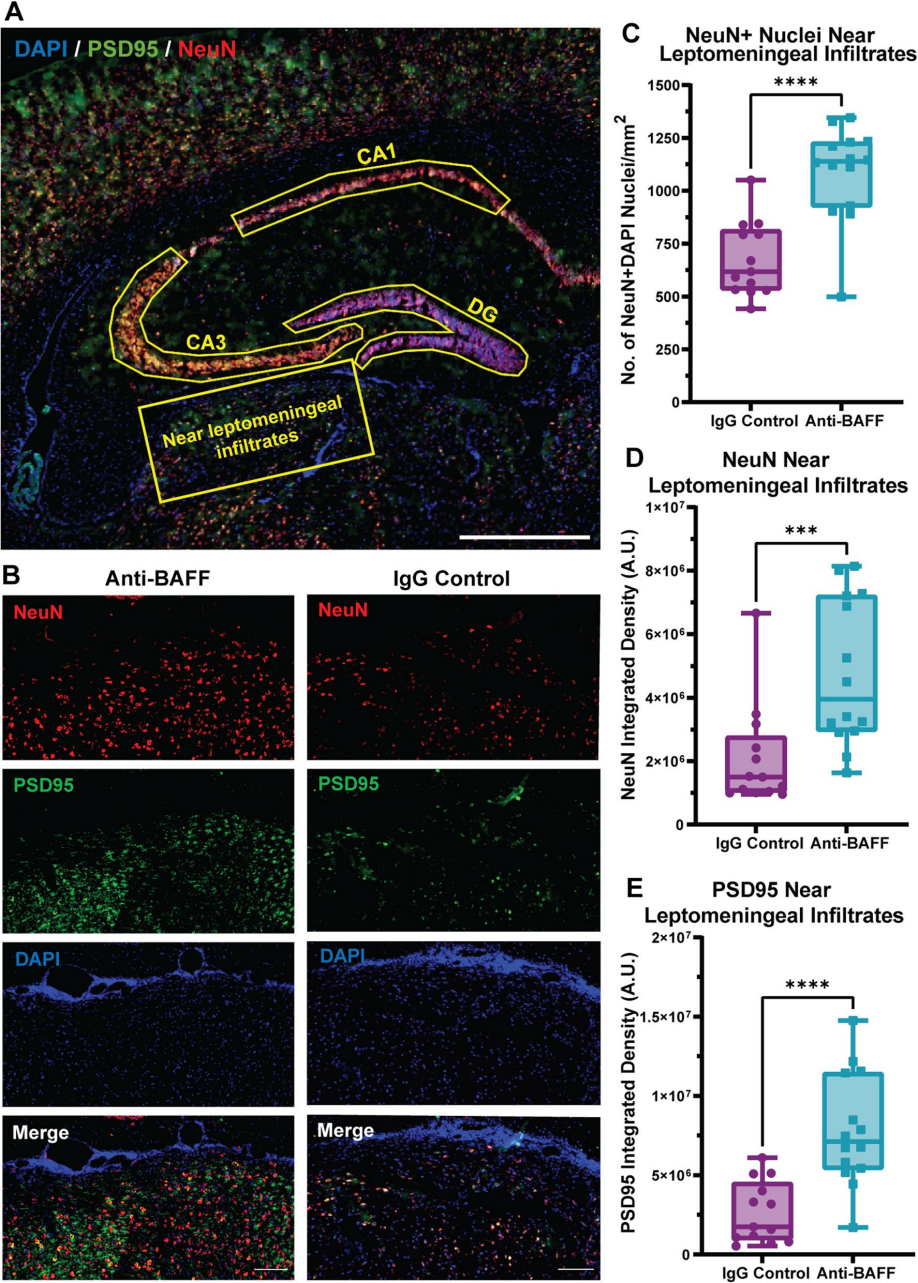
**BAFF blockade with anti-BAFF antibody 10F4 protects neurons and synapses near the meningeal infiltrates in rr-EAE.** **A** Overview of PSD95 (green), NeuN (red), and DAPI (blue) stained coronal section of brain from EAE mice representing the ROI of brain parenchyma near leptomeningeal infiltrates used for quantification of PSD95 and NeuN integrated density and to count the NeuN + DAPI nuclei to compare between the anti-BAFF antibody and control IgG-treated EAE mice. **B** Representative images of the brain parenchyma near leptomeningeal infiltrates stained with PSD95 and NeuN from anti-BAFF treated, and IgG control-treated EAE mice brain coronal section. **C** Quantification shows significantly higher number of NeuN + DAPI nuclei in the cortex near leptomeningeal infiltrates in anti-BAFF antibody-treated group. **D**, **E** Quantitative analysis shows the significantly higher integrated density of PSD95 and NeuN in the cortex near leptomeningeal infiltrates in anti-BAFF antibody-treated group. **C**, **D**, **E** The box plots with the center line indicating the median, the box indicating the 25th and 75th percentiles, the whiskers indicating range, and the dots representing all data points. The statistical analysis was conducted using the Mann–Whitney *U* test, and *P*-values were derived from it. The significance level was set to *P < 0.05, **P < 0.01, ***P < 0.001. The scale bars for the images shown were 100 um. The number of EAE mice for the anti-BAFF antibody 10F4 group was 14 and for the IgG control group was 13

We observed significantly higher synaptic density (as measured by the integrated density of synaptic protein PSD95) within the hippocampal regions (CA1, CA3, and DG) of EAE mice treated with the anti-BAFF antibody, as compared to those treated with the control IgG (Fig. 5B, C, D, F, H, J). The integrated density of NeuN which stains neuronal nuclei was also significantly higher in all hippocampal regions in the anti-BAFF treated group (Fig. 5B, C, D, E, G, I). Furthermore, we observed significant protection of NeuN-positive nuclei in the cortex adjacent to the meningeal infiltrates (Fig. 6B–D). Taken together, these results demonstrate the potential of anti-BAFF antibody mediated BAFF blockade in protecting synapses and neurons within the hippocampus and near the meningeal infiltrate in rr-EAE mice.

## Discussion

The relationship between leptomeningeal inflammation and severity and progression of MS suggests that this process may be a promising therapeutic target. Our study shows that in an rr-EAE model, anti-BAFF antibody therapy impacts meningeal inflammation in comparison to an IgG control antibody. This is evidenced by significant reductions of B cells (B220 +), T cells (CD3 +), and myeloid cells (Mac2 +) in the meninges of the anti-BAFF treated mice. Moreover, in the cortex adjacent to the regions of leptomeningeal inflammation and hippocampi of the anti-BAFF-treated mice there was a greater preservation of neurons and synapses.

Our previous work testing inhibition of Bruton tyrosine kinase (BTK), which is a non-receptor kinase that promotes B cell and myeloid cell activation and pro-inflammatory polarization, also showed similar reduction of immune cell infiltration into the meninges. Studies have shown that these cells, found in ectopic lymphoid follicles in the meninges of patients with MS, are linked to worse cortical pathology, higher severity, and earlier onset of disease [4, 6, 7]. In vitro studies have demonstrated that B cells from patients with MS cause cell death in neurons and oligodendrocytes via the release of cytotoxic factors [25]. This release may be mediated by extracellular vesicles produced by B cells that carry pro-inflammatory factors that promote cell death. Furthermore, they may lead to increased inflammation through recruitment and activation of microglia and astrocytes to cortical regions adjacent to meningeal inflammation. These studies point to the pathogenic role of B cell aggregates in meningeal inflammation and the course of MS disease, and consequently, the elimination of these cells as a potential therapeutic target.

BAFF binds with highest affinity to TNF receptor superfamily member 13C (more commonly known as BAFF-R), and with lower affinities to B cell maturation antigen (BCMA) and transmembrane activator and cyclophilin ligand interactor (TACI) [8, 26]. These interactions lead to B cell maturation and T cell stimulation, therefore, BAFF blockade in our study led to reduction in their aggregation in the meninges, which in turn may have led to better preservation of neurons and synapses in the neighboring cortex and hippocampus.

Another possible mechanism of action leading to anti-BAFF’s neuroprotective effects may be the inhibition of BAFF interaction with NgR receptors. These receptors bind with high affinity to myelin associated inhibitory factor (MAIF) Nogo-A, which inhibits axonal regeneration [27]. However, in EAE mouse models, BAFF-mediated NgR1 and NgR3 activation has been demonstrated to stimulate maturation of leptomeningeal B cells and actively promote axonal demyelination through secretion of IgG anti-myelin antibodies by NgR1^+^ and Ngr3^+^ B cells [12]. BAFF inhibition of these NgR receptors in the rr-EAE mice may have thus played a role in the reduction of synaptic and neuronal loss in the cortex.

Though we saw a reduction in the number and activation of immune cells in both the meninges and cortex, we did not see a reduction in astrocytosis near regions of LMI. One explanation for this may be that the astrocytosis is upstream of the BAFF blockade as BAFF in endogenously produced in the CNS by astrocytes [11]. However, BAFF inhibition was still effective in reducing the presence of other immune cell populations.

In our MRIs, we saw a trend towards increased volume of leptomeningeal enhancement in IgG control-treated mice over the course of disease, compared to anti-BAFF treated mice, which did not show this enlargement over time. Though this difference did not show a statistically significant interactive effect, it is possible that increasing the period of observation and conducting more MRIs after week 10 post-immunization could lead to a better understanding of the accumulation of meningeal infiltrates over the course of multiple EAE relapses. If the current diverging trend continues, we may find that anti-BAFF treatment significantly prevents worsening of leptomeningeal inflammation over the course of the disease.

One previous study examined the effect of a BCMA-Fc agent in NOD or C57/BL6 mice with EAE induced with whole myelin oligodendrocyte glycoprotein (MOG) immunization [28]. This study showed that the agent administered in a prevention paradigm reduced the incidence and severity of EAE and in a treatment paradigm, with treatment started early in the disease course there was a mild–moderate impact on behavioral scores. In contrast to this study, we did not note any significant difference in clinical scores during the 4-week treatment period despite the effects noted on leptomeningeal inflammation on histopathology. This may be due to the fact that clinical scores in the rr-EAE mice are primarily driven by spinal cord involvement and hence changes in inflammation in the meninges and effects on the cortex may not be captured by this outcome. Possibilities for the divergence in outcomes between our study and the prior study are—(1) the BCMA-Fc agent tested in the previous study would be expected to neutralize both BAFF and APRIL while in our study we utilized an antibody solely targeting BAFF; (2) whole MOG immunization induced EAE pathology has a greater contribution from B cells; and (3) the initiation of treatment earlier in the disease course in the prior study with a much longer treatment duration (almost 7–8 weeks).

BAFF blockade using anti-BAFF antibody 10F4 has also been studied in other immune-mediated disorders to good effect. Belimumab (the human analog of 10F4) is already an approved treatment for systemic lupus erythematosus (SLE). BAFF neutralization has also been shown to be a successful treatment for autoimmune diabetes in nonobese mice [23].

There are some important limitations to consider which include our inability to conclude whether reductions in immune cells in the meninges, especially B cells are due to reduced recruitment or survival (since BAFF is important for B cell survival and maturation). Additionally, we cannot definitively conclude whether reduced NeuN staining represents neuronal loss or stressed neurons. Given our results, it is also likely that longer treatment or perhaps additional dosing of BAFF antibody may have been of interest and these would be important in follow-up studies. Also, the effects on the cortex adjacent to areas of meningeal inflammation cannot be causatively linked to the treatment and could also represent more global effects of the anti-BAFF treatment. Lastly, with regard to our imaging outcomes, examining LME in the hindbrain may also have provided additional insight into the effect of BAFF on meningeal inflammation, however our imaging protocol to detect LME was optimized for the forebrain (largely to limit scanning time for sick mice) and we did not obtain slices through the hindbrain and hence cannot definitively comment on whether the changes in LME would have been similar in this region.

The success of anti-BAFF treatment in reducing meningeal inflammation and preventing cortical neuronal damage in our study and in treating other autoimmune diseases warrant further study of its efficacy as a potential therapy for MS.

## Conclusion

In this study, we demonstrate that BAFF blockade in mice with rr-EAE leads to a reduction in immune cells including B cells, T cells, and myeloid cells in meningeal infiltrates. Furthermore, BAFF blockade leads to the preservation of neurons and synaptic densities in the hippocampus and cortex adjacent to regions of meningeal inflammation. This reduction of inflammation and cortical pathology of EAE compared to that of the IgG control suggests that anti-BAFF therapy may be a novel avenue for treatment of MS.

### Supplementary Information

**Additional file 1: Figure S1. Clinical EAE Scores post treatment.** (A) The mean of daily EAE scores after starting treatment (post-Week 6 MRI) for the anti-BAFF treatment group and IgG control group showed no difference according to a mixed-effects analysis. P = 0.4271 for interaction effects. (B) There was also no significant difference in the mean of the cumulative EAE scores of each mouse in between the two groups, as represented by this box and whisker plot which shows the distribution of the cumulative EAE scores of each mouse post treatment (the center line indicates median, the box 25th–75th percentile of data, and the whiskers represent the range). P = 0.9223 using a Mann–Whitney U-test. **Figure S2: MRI leptomeningeal enhancement quantification method.** (A) Contrast enhancement in the same brain region in consecutive slides was considered as a single region of leptomeningeal inflammation and its volume was calculated across multiple slides using the quantification formula described in the methods section. Total LME Volume for each mouse was calculated by adding the volume of regions of leptomeningeal inflammation. These volumes were then averaged by treatment group for each MRI time point for data analysis.

## Data Availability

All data generated or analyzed during this study are included in this published article (and its Additional files) or are available on request from the corresponding author.

## Abbreviations

MS: Multiple sclerosis
EAE: Experimental autoimmune encephalomyelitis
rr-EAE: Relapsing–remitting experimental autoimmune encephalomyelitis
CNS: Central nervous system
MRI: Magnetic resonance imaging
LMI: Leptomeningeal inflammation
LME: Leptomeningeal enhancements (in MRI scans)
BAFF: B cell activating factor of the TNF family
APRIL: A-proliferation-inducing ligand
BAFF-R: BAFF receptor
TACI: Transmembrane activator and cyclophilin ligand interactor
BCMA: B cell maturation antigen
NgR: Nogo receptor
MAIF: Myelin associated inhibitory factor
NGS: Normal goat serum
DG: Dentate gyrus
BTK: Bruton tyrosine kinase
SLE: Systemic lupus erythematosus
