# Supplementary material for: BAFF blockade in experimental autoimmune encephalomyelitis reduces inflammation in the meninges and synaptic and neuronal loss in adjacent brain regions
Source: J Neuroinflammation. 2023 Oct 7;20:229. doi: 10.1186/s12974-023-02922-7 (PMC10559498; doi:10.1186/s12974-023-02922-7)
Supplement: Supplementary file 1 — Additional file 1: Figure S1. Clinical EAE Scores post treatment. (A) The mean of daily EAE scores after starting treatment (post-Week 6 MRI) for the anti-BAFF treatment group and IgG control group showed no difference according to a mixed-effects analysis. P = 0.4271 for interaction effects. (B) There was also no significant difference in the mean of the cumulative EAE scores of each mouse in between the two groups, as represented by this box and whisker plot which shows the distribution of the cumulative EAE scores of each mouse post treatment (the center line indicates median, the box 25th–75th percentile of data, and the whiskers represent the range). P = 0.9223 using a Mann–Whitney U-test. Figure S2: MRI leptomeningeal enhancement quantification method. (A) Contrast enhancement in the same brain region in consecutive slides was considered as a single region of leptomeningeal inflammation and its volume was calculated across multiple slides using the quantification formula described in the methods section. Total LME Volume for each mouse was calculated by adding the volume of regions of leptomeningeal inflammation. These volumes were then averaged by treatment group for each MRI time point for data analysis. [file 12974_2023_2922_MOESM1_ESM.docx]

**Additional file 1**

**c
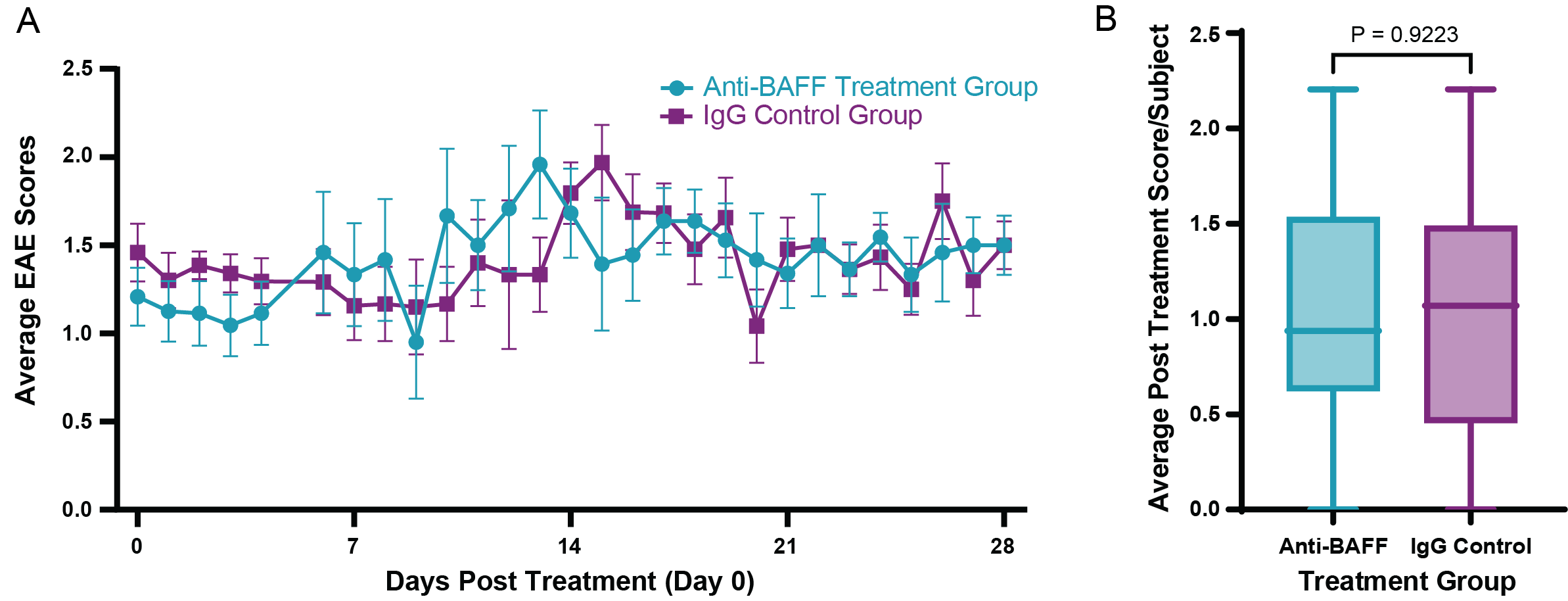
**

***Figure S1: Clinical EAE Scores post treatment.*** *(A) The mean of daily EAE scores after starting treatment (post Week 6 MRI) for the anti-BAFF treatment group and IgG control group showed no difference according to a mixed-effects analysis. P = 0.4271 for interaction effects. (B) There was also no significant difference in the mean of the cumulative EAE scores of each mouse in between the two groups, as represented by this box and whisker plot which shows the distribution of the cumulative EAE scores of each mouse post treatment (the center line indicates median, the box 25^th^-75^th^ percentile of data, and the whiskers represent the range). P = 0.9223 using a Mann-Whitney U-test.*

**
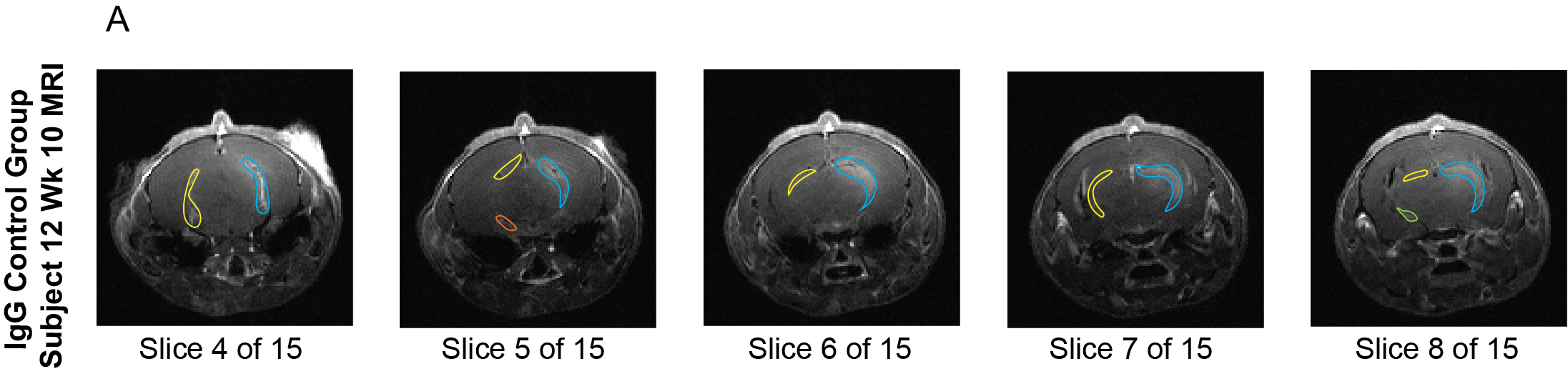
**

***Figure S2: MRI leptomeningeal enhancement quantification method.*** *(A) Contrast enhancement in the same brain region in consecutive slides was considered as a single region of leptomeningeal inflammation and its volume was calculated across multiple slides using the quantification formula described in the methods section. Total LME Volume for each mouse was calculated by adding the volume of regions of leptomeningeal inflammation. These volumes were then averaged by treatment group for each MRI time point for data analysis.*
